# *Zfhx3* modulates retinal sensitivity and circadian responses to light

**DOI:** 10.1096/fj.202100563R

**Published:** 2021-08-12

**Authors:** Steven Hughes, Jessica K. Edwards, Ashleigh G. Wilcox, Carina A. Pothecary, Alun R. Barnard, Russell Joynson, Greg Joynson, Mark W. Hankins, Stuart N. Peirson, Gareth Banks, Patrick M. Nolan

**Affiliations:** ^1^ Nuffield Department of Clinical Neurosciences Sir William Dunn School of Pathology Sleep and Circadian Neuroscience Institute University of Oxford Oxford UK; ^2^ MRC Harwell Institute Oxfordshire UK; ^3^ Nuffield Laboratory of Ophthalmology Department of Clinical Neurosciences University of Oxford Oxford UK

**Keywords:** amacrine cell, light sensitivity, mutation, pleiotropy, pupillary reflex

## Abstract

Mutations in transcription factors often exhibit pleiotropic effects related to their complex expression patterns and multiple regulatory targets. One such mutation in the zinc finger homeobox 3 (ZFHX3) transcription factor, short circuit (*Sci*, *Zfhx3^Sci/+^*), is associated with significant circadian deficits in mice. However, given evidence of its retinal expression, we set out to establish the effects of the mutation on retinal function using molecular, cellular, behavioral and electrophysiological measures. Immunohistochemistry confirms the expression of ZFHX3 in multiple retinal cell types, including GABAergic amacrine cells and retinal ganglion cells including intrinsically photosensitive retinal ganglion cells (ipRGCs). *Zfhx3^Sci/+^* mutants display reduced light responsiveness in locomotor activity and circadian entrainment, relatively normal electroretinogram and optomotor responses but exhibit an unexpected pupillary reflex phenotype with markedly increased sensitivity. Furthermore, multiple electrode array recordings of *Zfhx3^Sci/+^* retina show an increased sensitivity of ipRGC light responses.

**Abbreviations**
DD: constant darkness
ERG: electroretinogram
EYFP: enhanced yellow fluorescent protein
GCL: ganglion cell layer
INL: inner nuclear layer
IPL: inner plexiform layer
ipRGC: intrinsically photosensitive retinal ganglion cell
ISI: inter stimulus interval
LD: light:dark
LL: constant light
MEA: multiple electrode array
ONL: outer nuclear layer
OPL: outer plexiform layer
PLR: pupillary light response
RGC: retinal ganglion cell
VEP: visual evoked potential
ZT: zeitgeber time

## 1 INTRODUCTION

Mechanisms through which the retinal circuitry relays photic information to the mammalian brain include both classical visual pathways as well as accessory visual responses, including regulation of circadian physiology and behavior. Many elements of the cell autonomous circadian regulatory mechanism contribute to circadian function in the retina. The mammalian retina contains an autonomous circadian clock and its function is believed to lie in gating physiological responses to the daily variations in the light/dark cycle.^1^, ^2^ Aside from elements that comprise the autonomous clock, additional genes and mechanisms, specifically those associated with region‐specific transcription factors, can impact upon the relay of environmental light cues to the brain.^3^, ^4^ Typically, these factors are critical in cell fate specification while their continued high levels of expression in adult tissues suggest that they retain function in terminally differentiated cells. Mutations in these genes are often associated with pleiotropy indicating that the genes can serve multiple roles in distinct cells and tissues.^5^

The transcription factor zinc finger homeobox 3, *Zfhx3*, is one of a number of transcription factors highly expressed during discrete periods in nervous system development whilst retaining high expression in a number of discrete adult brain nuclei including the suprachiasmatic nucleus (SCN) of the hypothalamus. Through the analysis of discrete mutations in mice, *Zfhx3* has been shown to have important roles in both the development and maintenance of SCN circadian pacemaker function.^6^, ^7^, ^8^ A dominant mis‐sense mutation in *Zfhx3* (short circuit, *Zfhx3^Sci/+^*) expresses a short circadian free‐running period in constant darkness and gives rise to altered expression of neuropeptide neurotransmitters and receptors critical for intercellular signaling within the SCN.^7^ These effects are not purely dependent on developmental expression of *Zfhx3*. Specific inducible deletion of *Zfhx3* in adult mice acutely shortens wheel‐running period or causes arrhythmicity and accelerates re‐entrainment to a 6‐h phase advance in the light dark cycle.^8^

In this study, we use a combination of behavioral, cellular expression and electrophysiological approaches to demonstrate that, in addition to important roles in SCN function, ZFHX3 likely also performs further direct roles in retinal function. Systematic analysis of *Zfhx3^Sci/+^* animals shows a range of abnormal responses to light stimuli at the level of the pupillary light reflex, cortical responses and in overt behaviors seen in circadian wheel‐running screens while optomotor responses are relatively normal. Immunohistochemistry confirms the expression of ZFHX3 within GABAergic amacrine cells, and also a subset of RGCs that includes melanopsin expressing intrinsically photosensitive retinal ganglion cells (ipRGCs). Expression of the mutant *Zfhx3^Sci/+^* allele in these cell types could account for the diversity in behavioral light responses in mutant animals. At an electrophysiological level, retinas from mutant animals have relatively normal electroretinograms (ERG) while multiple electrode array recordings of *Zfhx3^Sci/+^* mutant retina shows an increased sensitivity and prolonged duration of ipRGC light responses.

Collectively, our data show that ZFHX3 is a transcription factor with biologically relevant roles not just in the SCN but also in the retina, with a potential to influence both visual and non‐visual responses to light including photic input to the SCN. These data provide new mechanisms by which ZFHX3 likely influences circadian entrainment pathways and highlight the need to consider retinal phenotypes in animal models with altered circadian function attributed to central SCN dysregulation.

## 2 MATERIALS AND METHODS

### 2.1 Animals

All procedures were conducted in accordance with the UK Home Office Animals (Scientific Procedures) Act 1986 (Project Licenses 30/2686, 30/2812, 30/3206 and 30/3371). All experiments were approved by local Ethical Review Board and performed in accordance with the MRC Harwell and University of Oxford policy on the use of animals in scientific research. *Zfhx3^Sci/+^* mice, carrying a mis‐sense mutation in the *Zfhx3* gene,^7^ were generated and bred at MRC Harwell and were maintained on a mixed C3H/HeH × C57BL6/J F1 background. Colonies were bred and housed in individually ventilated cages (IVCs) in a pathogen‐free environment. Unless stated otherwise, all mice were kept under a 12:12 light dark cycle with food and water provided ad libitum. *Zfhx3^Sci/+^* animals were genotyped from single ear biopsies using a real time PCR (RT‐PCR) Taqman assay with the following primers and probes (5′ to 3′): FW TCCACGCATTGCTTCAGATG. REV TGTGCCTTCTGCTTGTTCTCA. Mutant probe VIC—TTTGAGCTCTTCATTCA. Wildtype probe FAM—CTTTGAGCTCGTCATT. As the C3H/HeH strain carries a recessive mutation resulting in retinal degeneration and loss of rod and cone photoreceptors (*Pde6b^rd1/rd1^*), test cohorts for these studies were genotyped for *Pde6b* and animals homozygous for the recessive mutant allele were omitted from the study. For co‐localization studies, ocular tissue was collected from *Opn4* ^+/−^tau‐*LacZ* ^+/−^ mice (mixed C57BL/6 and 129/SvJ background) that express a βgal reporter in M1 type ipRGCs^9^ and heterozygous *Opn4* ^+/−^ *Cre* ^+/−^ *EYFP* ^+/+^ mice (mixed C57BL/6 and 129/SvJ background) that express EYFP within M1‐M5 type ipRGCs.^10^, ^11^

### 2.2 Circadian screening

Mice from 8 weeks of age were individually housed in cages within light‐tight chambers, kept under environmentally controlled conditions with food and water provided ad libitum. Revolutions of running wheels fitted into cages were monitored using Clocklab (Actimetrics) and plotted as activity on double‐plotted actograms.^12^ Mice were initially held under a 12:12 light‐dark cycle for a 1‐week entrainment period, followed by two weeks of constant darkness (DD) and then two weeks of constant light (LL), to assess free‐running activity. A fluorescent light source was used for all screens, with light intensities varied from 0.1‐100 lux. In a separate protocol following 7‐10 days in a 100 lux LD cycle, mice were switched to a reduced intensity LD cycle (10 lux or 1 lux) while simultaneously delaying the phase of the LD cycle by 2 hours. Mice were maintained in this altered schedule until the phase angle of entrainment could be determined and the returned to the default LD schedule.

### 2.3 Optomotor response

Male mice aged 10‐12 weeks were tested for optomotor responses using a visual tracking drum, OptoMotry system (CerebralMechanics) as described previously.^13^ The test arena comprised a striped image projected by four interfacing LCD monitors and rotating in one direction. Mice were individually placed onto a raised circular platform of 8 cm diameter in the center of the four screens. The frequencies of stripes used were 0.25 cycles/degree (subtending an angle of 2° when viewed from the center of the drum), 0.125 cycles/degree (4°), or 0.0625 cycles/degree (8°). The pattern was rotated anticlockwise for 30 seconds at two revolutions per minute (12°/sec) and head tracking response used to assess right eye ability, and then repeated with clockwise rotation to assess left eye ability. Tests started with a 2° stripe and if no head tracking response was observed, the stripe was increased to 4° and 8°. Visual acuity was assessed as the frequency where head tracking was evident.

### 2.4 Pupillometry

Measurements of the pupillary light response were carried out as previously described.^13^ Animals were housed on a 12:12 light‐dark cycle and tested between zeitgeber time (ZT) 4 and 8. Animals were dark adapted for 1‐2 hours prior to testing. A xenon arc lamp (150 W solar simulator, Lot Oriel, UK) with a 480 nm monochromatic filter (Andover, 10 nm half‐bandwidth) was used to produce light stimuli ranging in intensity from 9.1‐14.6 log quanta/cm^2^/s (173 µW/cm^2^/s). Light was transmitted to the eye via a liquid light pipe as an irradiant light stimulus using a 5 cm integrating sphere (Pro‐lite Technology, UK). Images of the consensual pupil responses were collected with a Prosilica near infrared sensitive charge couple device video camera (Allied Vision, Stadtroda, Germany) at a rate of 10 frames/s, positioned perpendicular to the contra‐lateral eye, which was illuminated by infra‐red light emitting diodes (850 nm, 10 nm half‐bandwidth). Five minutes prior to recording, 1% tropicamide was applied to the stimulated eye. Animals were temporarily restrained by “scruffing”, using normal husbandry techniques for the duration of the recording (29 seconds). After brief baseline measurements of the dark‐adapted pupil (2 seconds), the left eye was exposed to the light stimulus for 10 seconds. Recovery data were collected for a post‐stimulus period of 17 seconds. Each animal was tested on multiple occasions to minimize any artifacts due to handling. Images were analyzed using ImageJ (http://rsbweb.nih.gov/ij/).

### 2.5 Immunocytochemistry

Collection, fixation and immunohistochemistry of retinal samples was performed as described previously.^11^, ^14^ Retinal sections at 18µm were permeabilized in PBS with 0.2% Triton‐X for 20 minutes at room temperature, blocked in PBS with 0.2% Triton‐X and 10% donkey serum (Sigma) for 1hr. Primary and secondary antibodies (details in Table S1) were diluted in PBS with 2.5% donkey serum and 0.2% Triton‐X and incubated for 16‐72 hours at 4°C and 2 hours at RT respectively. Slides were mounted with Prolong Gold anti‐fade reagent containing DAPI nuclear counter stain (Life Technologies). Similar protocols were used for staining of retina whole mounts, although levels of Triton‐X in buffers were increased to 1% for permeabilization, blocking steps, and incubation with primary and secondary antibodies. Fluorescent images were acquired using an inverted LSM 710 laser scanning confocal microscope (Zeiss) with Zen 2010 image acquisition software (Zeiss) with excitation at 405, 488 and 561 nm and emission at 440‐480 nm, 505‐550 nm and 600‐700 nm for DAPI, green and red fluorescence respectively. Images were collected every 1µm in the z‐plane and merged to form full‐depth images. Global enhancements of brightness and contrast were performed using Zen 2010 software.

### 2.6 Histology

Cryopreserved 12 μm tissue sections were stained with hematoxylin and eosin using a Shandon Varistain 24 auto‐stainer. Slides were mounted in glycerol and visualized by light microscopy.

### 2.7 qPCR analysis

Immediately following enucleation, retinas were dissected and snap frozen on dry‐ice and stored at −80°C. RNA extraction was performed using Trizol reagent (Thermo Fisher), and the concentration of RNA assessed using a Nanodrop ND1000 (Isogen Life Science). cDNA was prepared from the template RNA using SuperScript™ III First Strand cDNA Synthesis SuperMix (Thermo Fisher). Each qPCR reaction was performed in triplicate using AB Fast Mastermix (Thermo Fisher) and an AB 7500 Fast PCR machine (Thermo Fisher). Analysis of amplification data was performed with AB software, employing a ddCt method using the *Rpli3a* gene as the endogenous control. Primer sequences are shown in supplementary material (Table S2).

### 2.8 In vivo electrophysiology

Electroretinogram (ERG) and flash visual evoked potential (VEP) responses were conducted as described previously.^15^ Before testing, mice were dark‐adapted overnight (>16 hours) and all experimental preparation was performed under dim red illumination. For the procedure, animals were anaesthetized and pupils were fully dilated with 1% tropicamide and 2.5% phenylephrine hydrochloride eye drops (Bausch & Lomb, UK). Animals were placed on a heated platform for the duration of the procedure. For the ERG active electrode, custom‐made contact lens electrodes were placed on both eyes with a 1% methylcellulose solution used as the coupling agent. For the VEP active electrode, a platinum needle (Grass technologies Inc) was placed subcutaneous in the scalp, approximately 2 mm lateral to lambda, overlying a large area of the right visual cortex. Subcutaneous platinum needles in the face and flank served as reference and ground electrodes, respectively. The platform was then positioned within a colordome light stimulator connected to an Espion E3 console (both Diagnosys LLC, Cambridge, UK). The Espion console differentially amplified and digitized signals recorded from the electrodes at a rate of 5 kHz and controlled the delivery of the light stimuli. All recordings were made in a custom‐made, light‐tight Faraday cage. For dark‐adapted testing, responses were elicited by brief (4 ms), single flashes of white light on a dark background. Stimulus intensity was increased across a 7 log unit range (−6 to 1 log cd s/m^2^) in log unit steps with the following response averaging and interstimulus interval (ISI) times used: −6 to −5 log cd s/m^2^, 16 responses averaged with an ISI of 3 seconds; −4 to −3 log cd s/m^2^, 9 responses, ISI = 4 seconds; −2 log cd s/m^2^, 9 responses, ISI = 8 seconds; −1 log cd s/m^2^, 4 responses, ISI = 16 seconds; 0 log cd s/m^2^, 4 responses, ISI = 32 seconds; 1 log cd s/m^2^, 4 responses, ISI = 64 seconds. Animals were then exposed to a full field 30 cd/m^2^ white background for at least 10 minutes (light‐adapted). Responses were then recorded to brief light flashes of three intensities (0, 1 & 1.4 log cd s/m^2^) superimposed on the stable background. In all cases, an ISI of 1 second was used and 50 responses were averaged per result. The amplitude and latency of the major ERG components (a‐ and b‐waves for dark‐adapted, b‐wave only for light‐adapted) was measured using automated and manual methods. By convention, b‐wave amplitude is measured from the a‐wave trough (where present) and a‐wave amplitude is measured from baseline to a‐wave trough. The amplitude and timing of the major ERG and VEP components was measured with the Espion software (Diagnosys LLC, Cambridge, UK) using automated and manual methods, without knowledge of the animal's genotype.

### 2.9 Multiple electrode array (MEA) recordings

Multi‐electrode array (MEA) recordings were performed as described previously.^16^, ^17^ Dissected retinas were mounted ganglion cell‐side down onto MEA recording chambers with 60 electrodes (30 μm Ø, 200 μm apart, Multi Channel Systems) and placed into a recording amplifier (MEA2100‐2x60, Multi Channel Systems) positioned within the light path of an Olympus IX71 inverted microscope. Retinas were perfused continuously with AMES media 95% O_2_ 5% CO_2_ (pH 7.4) maintained at 34°C. Endogenous melanopsin based ipRGC light responses were isolated from rod and cone driven inputs using a cocktail of synaptic blockers^18^ 100 μm L(+)‐2‐amino‐4‐phosphonobutyrate (L‐AP4) (group III metabotropic glutamate receptor agonist), 40 μm 6,7‐dinitroquinoxaline‐2,3‐dione (DNQX) (AMPA/kainate receptor antagonist), and 30 μm d‐2‐amino‐5‐ phosphonovalerate (d‐AP5) (NMDA receptor antagonist) (Tocris). Light stimuli (480 nm, 10.1‐15.1 log quanta) were generated by an X‐cite 120W metal halide light source (EXFO) and 480 ± 10 nm band pass filter (Thor Labs) and controlled by a high‐speed shutter and automated filter wheel (Prior Scientific) containing neutral density filters (0 to 7 log units, Thor Labs). Power of light stimuli (µW/cm^2^/s) were measured at the sample focal plane using an in‐line power meter (PM160T, Thor Labs) and converted to photon flux (log quanta) using an irradiance conversion toolbox.^19^ Analysis of spike firing rates was performed using MC rack software (Multi Channel Systems), with a 200 Hz high pass filter and spike detection threshold of 4 standard deviations. Spikes from each electrode were collected into 1 second bins (spikes/s) and analyzed with MS Excel.

### 2.10 Data analysis and statistics

Unless otherwise stated, statistical differences were established using a Student's *t* test. Circadian analysis, optokinetic drum scores, pupillometry, morphological measures qPCR, VEP and ERG data were analyzed using GraphPad Prism (GraphPad Software). MEA data was analyzed using MS Excel (Microsoft). Significance level for all analysis was set at *P* < .05.

## 3 RESULTS

### 3.1 ZFHX3 is expressed in the inner nuclear and ganglion cell layers of mouse retina

**FIGURE 1:**
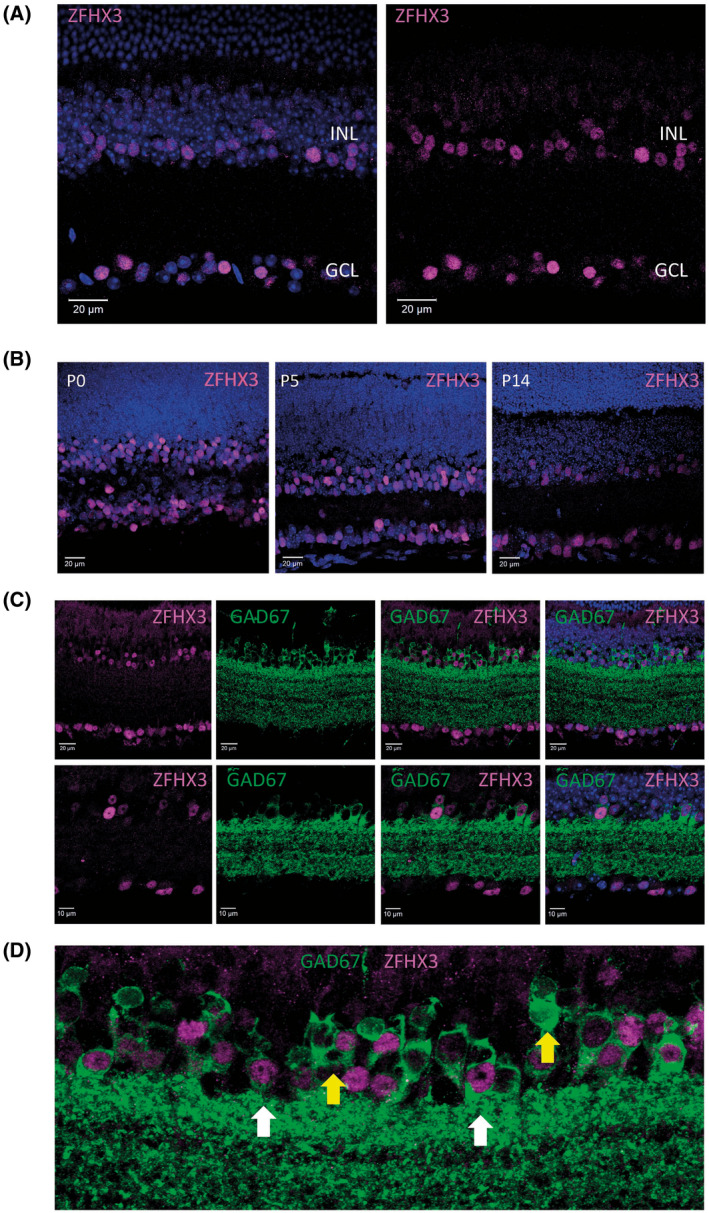
ZFHX3 is expressed in GAD67‐positive amacrine cells. A, Images from retina flat‐mounts (left) and retina sections showing the localization of ZFHX3 in adult mouse retina (age > P60), with ZFHX3 expressing cells located predominately on the inner surface of the INL and the GCL. B, Images showing the localization of ZFHX3 in the mouse retina throughout postnatal development (ages P0, P5 and P14), showing higher levels of ZFHX3 expression during early postnatal development. C, Images showing the co‐localization of ZFHX3 and the GABAergic amacrine cell marker GAD67 in adult mouse retina. D, Higher magnification of (C). ZFHX3 expression within the INL is predominately confined to GAD67 positive amacrine cells, with approximately 60% of GAD67 amacrine cells expressing detectable levels of ZFHX3. ZFHX3 expression was also detected within GAD67 positive amacrine cell located in the GCL. White arrows highlight examples of ZFHX3 expressing GAD67 amacrine cells. Yellow arrows show examples of GAD67 amacrine cells for which ZFHX3 was not detected. DAPI nuclear counter stain is shown in blue. INL, inner nuclear layer; IPL, inner plexiform layer; GCL, ganglion cell layer

Transcriptomic studies in the developing retina have suggested that ZFHX3 is expressed at least transiently in the retina.^20^ By immunostaining with a custom‐generated antibody to ZFHX3,^7^ we confirmed that ZFHX3 protein is expressed in both the developing and adult mouse retina, and is localized predominantly within the nucleus of cells located in the inner nuclear layer (INL) and ganglion cell layer (GCL) (Figure 1A,B). Levels of ZFHX3 expression are developmentally regulated in postnatal retinal tissue with levels of immunostaining and the number of immunoreactive cells higher at P0 compared to P5. Levels of ZFHX3 staining are further reduced by P14 and are comparable to those in mature adult tissue.

**FIGURE 2:**
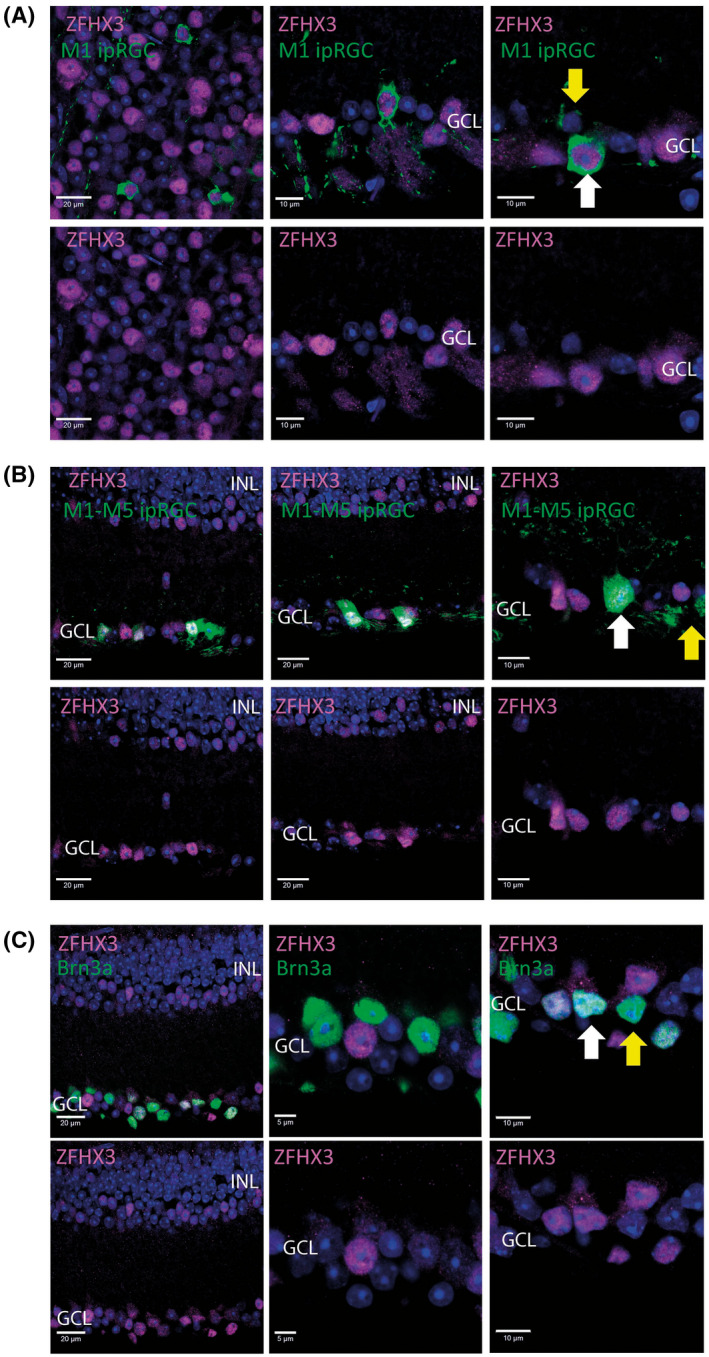
ZFHX3 is expressed in diverse retinal ganglion cell layer subtypes. A, Images showing the co‐localization of ZFHX3 and βgal in the retina of adult Opn4^+/−^Lacz^+/−^ mice showing consistent expression of ZFHX3 in M1 type ipRGCs. B, Images showing the co‐localization of ZFHX3 and eYFP in the retina of adult Opn4^+/−^Cre^+/−^eYFP^+/+^ mice showing expression of ZFHX3 in a subset of all ipRGCs (~40%), including cells with morphologies characteristic of non‐M1 ipRGCs. C, Images showing the co‐localization of ZFHX3 and the retinal ganglion cell marker BRN3A in adult mouse retina. White arrows highlight ZFHX3 positive cells. Yellow arrows show examples of cells for which ZFHX3 was not detected. DAPI nuclear counter stain is shown in blue. INL, inner nuclear layer; GCL, ganglion cell layer

Double immunofluorescence labelling was used to profile the retinal expression of ZFHX3 in the adult retina. Co‐staining with antibodies that recognize a range of major retinal cell types revealed that ZFHX3 INL expression is predominately within GAD67‐positive GABAergic amacrine cells (with ~60% of these cells showing convincing ZFHX3 expression) (Figure 1C,D). ZFHX3 was also found to be expressed in a range of retinal ganglion subtypes, including close to 100% of melanopsin expressing M1 type ipRGCs identified by βgal expression in the retina of *Opn4^+/−^LacZ^+/−^* mice that selectively reports M1 ipRGCs (Figure 2A), with lower levels of co‐localization observed for non‐M1 type ipRGCs characterized by stratification of dendrites in the ON layers of the inner plexiform layer and identified by eYFP expression in the retina of *Opn4* ^+/−^ *Cre* ^+/−^ *eYFP* mice that report all ipRGC subtypes (~20%) (Figure 2B) and, also, BRN3A positive RGCs (~20%, where BRN3A labels a diverse range of RGCs involved in classical visual pathways) (Figure 2C).

### 3.2 *Zfhx3^Sci/+^* mice show aberrant behavioral and physiological responses to light

**FIGURE 3:**
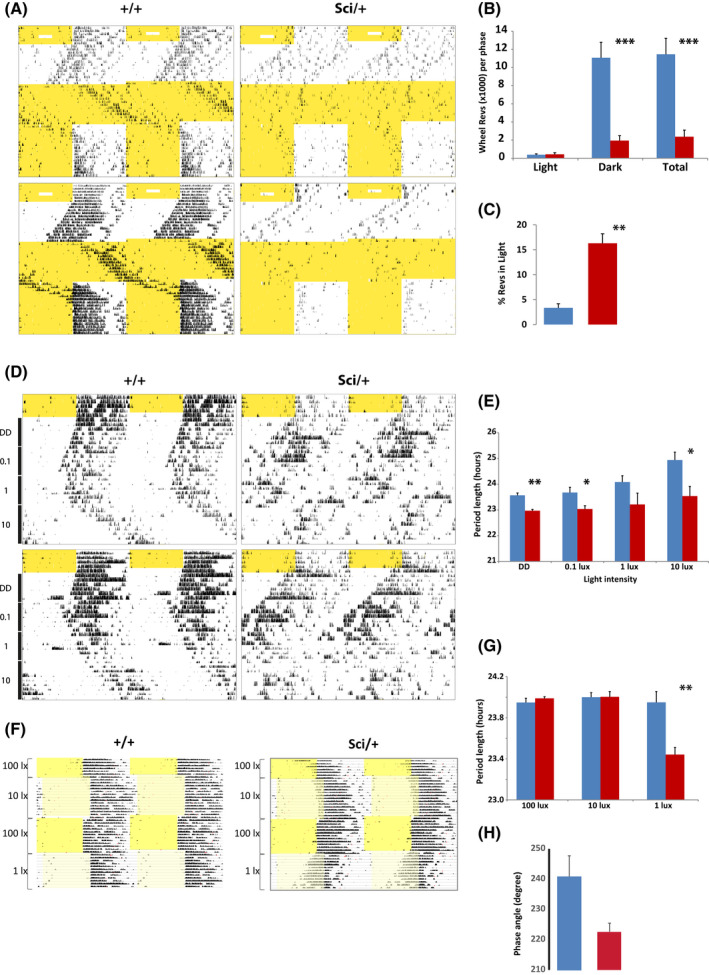
Wheel running parameters in *Zfhx3^Sci/+^* in altered light conditions and schedules. Two representative double‐plotted actograms for *Zfhx3^+/+^* and *Zfhx3^Sci/+^* animals (A). Animals were initially entrained to 12:12 light‐dark (LD) cycle for seven days, subjected to episodes of constant darkness (unshaded) and constant light (yellow shading) and subsequently returned to the original LD cycle. Shaded parts of actograms represent lights‐on, wheel‐running is represented as vertical black bars. B, Average wheel revolutions per phase under LD conditions for light phase, dark phase and entire 24‐hour interval in wild‐type (black, n = 6) and *Zfhx3^Sci/+^* (red, n = 9) animals. *Zfhx3^Sci/+^* animals show less robust wheel running overall but run more during the light phase. C, Percentage of total wheel revolutions in light phase for wild‐type (black) and *Zfhx3^Sci/+^* (red) animals. D, Two representative double‐plotted actograms of *Zfhx3^+/+^* and *Zfhx3^Sci/+^* animals maintained for 5 days in 12:12 LD conditions, for 7 days in constant darkness and subsequently in constant light of increasing intensity (0.1, 1 and 10 lux) for 7 days under each condition. Shaded parts of actograms represent lights‐on, wheel‐running is represented as vertical black bars. E, Period of activity onsets in constant darkness and in constant light conditions of increasing intensity in *Zfhx3^+/+^* (black, n = 6) and *Zfhx3^Sci/+^* (red, n = 6) animals. F, Representative double‐plotted actograms of *Zfhx3^+/+^* and *Zfhx3^Sci/+^* animals in 12:12 LD conditions. Animals were initially maintained in a light‐dark cycle of 100 lux. After 7 days, light intensity was reduced to 10 lux while simultaneously delaying the phase of the LD cycle by 4 hours. Animals were subsequently returned to 100 lux conditions with a simultaneous phase advance of 4 hours and, finally, light intensity was reduced to 1 lux, again with a simultaneous phase delay of 4 hours. Shaded parts of actograms represent lights‐on, wheel‐running is represented as vertical black bars. G, Period of activity onsets in bright and dim light conditions in *Zfhx3^+/+^* (black, n = 7) and *Zfhx3^Sci/+^* (red, n = 7) animals. H, Phase angle of entrainment in *Zfhx3^+/+^* and *Zfhx3^Sci/+^* animals in 12:12 LD conditions at a light intensity of 10 lux. All values are expressed as mean ± SEM (*P < .05, **P < .01, ***P < .001)

To investigate the functional significance of ZFHX3 expression in retina, we studied a range of light responsive functions in *Zfhx3^Sci/+^*, a mouse line carrying a point mutation in *Zfhx3*. In circadian wheel‐running studies, *Zfhx3^Sci/+^* mice display a characteristically short free‐running period under conditions of constant darkness (DD) (Figure 3A) consistent with that observed in previous studies.^7^ Moreover, *Zfhx3^Sci/+^* mice express variable phenotypes under 12:12 light‐dark cycles (LD) and under conditions of constant light (LL). Under LD conditions *Zfhx3^Sci/+^* mutants display significantly less overall activity compared to *Zfhx3^+/+^* controls (*P* = .0008) (Figure 3A,B) and a significantly greater proportion of activity in the light (rest) phase of the LD cycle (*P* = .005) (Figure 3C). Under LL conditions, behavioral responses were variable. While individual animals showed some period lengthening, the majority showed splitting of the circadian activity patterns and/or a tendency toward arrhythmic behavior (Figure 3A). Subsequently, on returning to a 12:12 LD cycle, *Zfhx3^Sci/+^* mice failed to appropriately re‐entrain, instead either continuing to free run or showing weak biphasic activity onsets around light‐dark transitions (Figure 3A).

Given the variable behavioral responses to light in LL conditions, we proceeded to investigate how the intensity of constant illumination affects the behavior of *Zfhx3^Sci/+^* mice by starting with DD and gradually increasing the intensity of LL conditions. Cohorts of mutant and control mice were allowed to free run for six days in DD conditions and subsequently for six days in LL conditions of increasing intensity (0.1, 1 and 10 lux, Figure 3D,E). Period lengthening behavior with increased light intensity was more consistently and robustly observed in the control animals from 1 lux (83% of animals) with lengthening in all animals at 10 lux. *Zfhx3^Sci/+^* animals showed more variable responses with only 33% animals showing a lengthening at either 1 or 10 lux. At these irradiances no lengthening was observed in 16.5% of animals while 33% showed split activity and 16.5% were arrhythmic. A significant difference was detected between the observed behaviors at both 0.1 and 10 lux (*P* < .05 at each irradiance level). A phase‐shifting protocol with simultaneous changes in light intensity was used to further assess entrainment, masking behavior and sensitivity to dim light in mutants (Figure 3F‐H). Animals were maintained in a 12:12 LD cycle while light intensity was reduced in successive steps from 100 lux ‐> 10 lux ‐> 100 lux ‐> 1 lux. The phase of the LD cycle was advanced or delayed by 4 hours at the same time as the reduction in light intensity. Transitions from 100 lux to 10 lux clearly showed that *Zfhx3^Sci/+^* mice did not re‐entrain to the altered LD phase (Figure 3F) although differences from controls in phase angle of entrainment were not significant (Figure 3H). However, chi‐squared analysis indicated a significant difference in the number of animals displaying normal entrainment (*χ* ^2^ = 6.3, 1 *df*, *P* = .012). Upon transition from 100 lux to 1 lux, the mutant phenotype is clearly evident as *Zfhx3^Sci/+^* start to free‐run immediately. A significant difference between *Zfhx3^Sci/+^* and wild‐type was detected when comparing period lengths at 1 lux (*P* = .004, Figure 3G).

**FIGURE 4:**
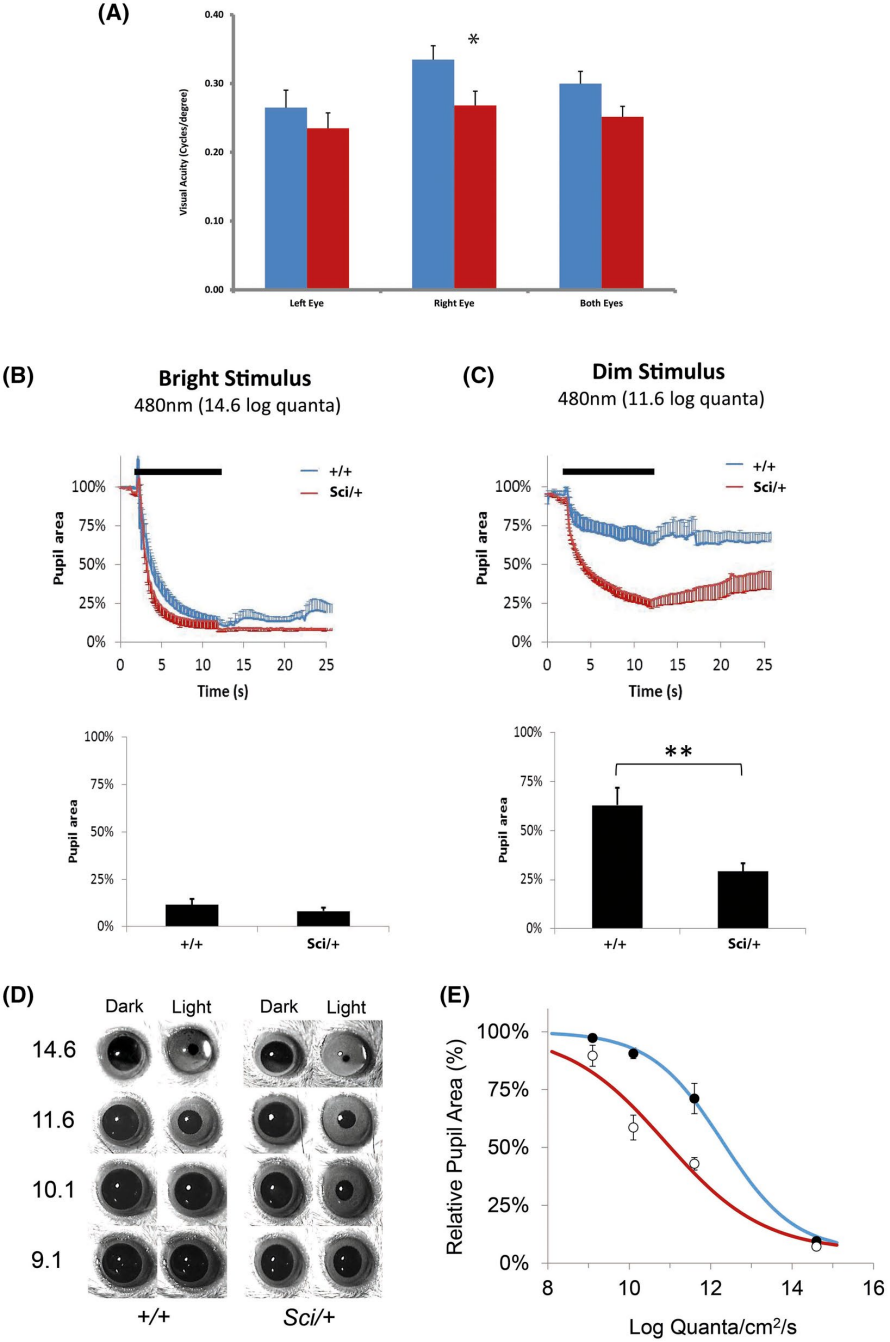
Visual and non‐visual retinal assessment in mice. Determination of visual acuity in *Zfhx3^+/+^* and *Zfhx3^Sci/+^* animals using the optokinetic drum and measurements of reflexive head tracking (A). A significant difference in the visual acuity was detected between *Zfhx3^+/+^* (blue, n = 9) and *Zfhx3^Sci/+^* (red, n = 8) animals when comparing the right eye individually and the averaged visual acuity of both the left and right eyes (*P* = .037 and *P* = .051 respectively). No significant differences were observed between *Zfhx3^+/+^* and *Zfhx3^Sci/+^* when assessing the visual acuity of the left eye individually. B, Consensual pupillary response. Top panel. Traces showing change in pupil area (% of baseline) over time during and after a bright light stimulus (14.6 log quanta/cm^2^/s) in *Zfhx3^+/+^* (blue, n = 7) and *Zfhx3^Sci/+^* (red, n = 8). Bottom panel. Maximal pupil constriction in response to a bright light stimulus is equivalent in *Zfhx3^+/+^* and *Zfhx3^Sci/+^* animals, with pupil area reduced to approximately 10% of dark‐adapted values. C, Top panel. Traces showing the change in pupil area (% of baseline) during and following exposure to a dim light stimulus (11.6 log quanta/cm^2^/s). *Zfhx3^Sci/+^* mice show an over constriction of the pupil in response to the dim light. Bottom panel. Maximal pupil constriction in response to a dim light stimulus. *Zfhx3^+/+^* animals constrict to approximately 60% whereas *Zfhx3^Sci/+^* animals constrict to approximately 25% (*P* = .0078). D, Representative images of the pupil area both before (Dark) and after (Light) stimulation for *Zfhx3^+/+^* and *Zfhx3^Sci/+^* animals. E, Relative percentage pupil area performed across four irradiances showing a shift in the irradiance response curve between *Zfhx3^+/+^* and *Zfhx3^Sci/+^* animals, equating to an approximately twenty‐fold increased sensitivity to light in mutants (1.36 log units). (*P < .05, **P < .01)

Unlike changes in free running periods under constant darkness which are directly associated with changes in SCN function and altered circadian rhythmicity, additional phenotypes observed in *Zfhx3^Sci/+^* mice in response to light stimuli are suggestive of additional effects at the level of the retina and altered photoentrainment pathways. We therefore conducted further investigations of ocular function in *Zfhx3^Sci/+^* animals. Visual assessment using the optomotor drum indicated only a marginal effect. For the right eye individually, and left and right eyes combined, *Zfhx3^Sci/+^* mice display decreased visual performance by 25% (right eye, *P* = .037) and 16% (combined, *P* = .051) although significance was borderline (Figure 4A). This trend was observed for the left eye although it was not significant (10.5% decrease, *P* = .394). Finally, to assess the physiological response of the retina to light, we measured the pupillary light response (PLR) in *Zfhx3^Sci/+^* mutant mice. By measuring the PLR to a range of decreasing light intensities, we set out to determine the relative sensitivities of *Zfhx3^Sci/+^* and control animals to light. At the brightest (14.6 log quanta/cm^2^/s) stimulus intensity, pupillary constriction was equivalent between control and *Zfhx3^Sci/+^* animals (Figure 4B,D). However, at 11.6 log quanta/cm^2^/s, *Zfhx3^Sci/+^* mice showed a significantly increased constriction of the pupil compared to control mice (*P* = .0078) (Figure 4C,D). Comparison of resulting irradiance response curves (IRC) plotted for *Zfhx3^Sci/+^* and *Zfhx3^+/+^* animals shows a shift of 1.36 log units, equating to an approximately twenty‐fold increased sensitivity of the PLR in *Zfhx3^Sci/+^* animals (Figure 4E).

### 3.3 Morphological assessment of anterior segment and retina of *Zfhx3^Sci/+^* mice

**FIGURE 5:**
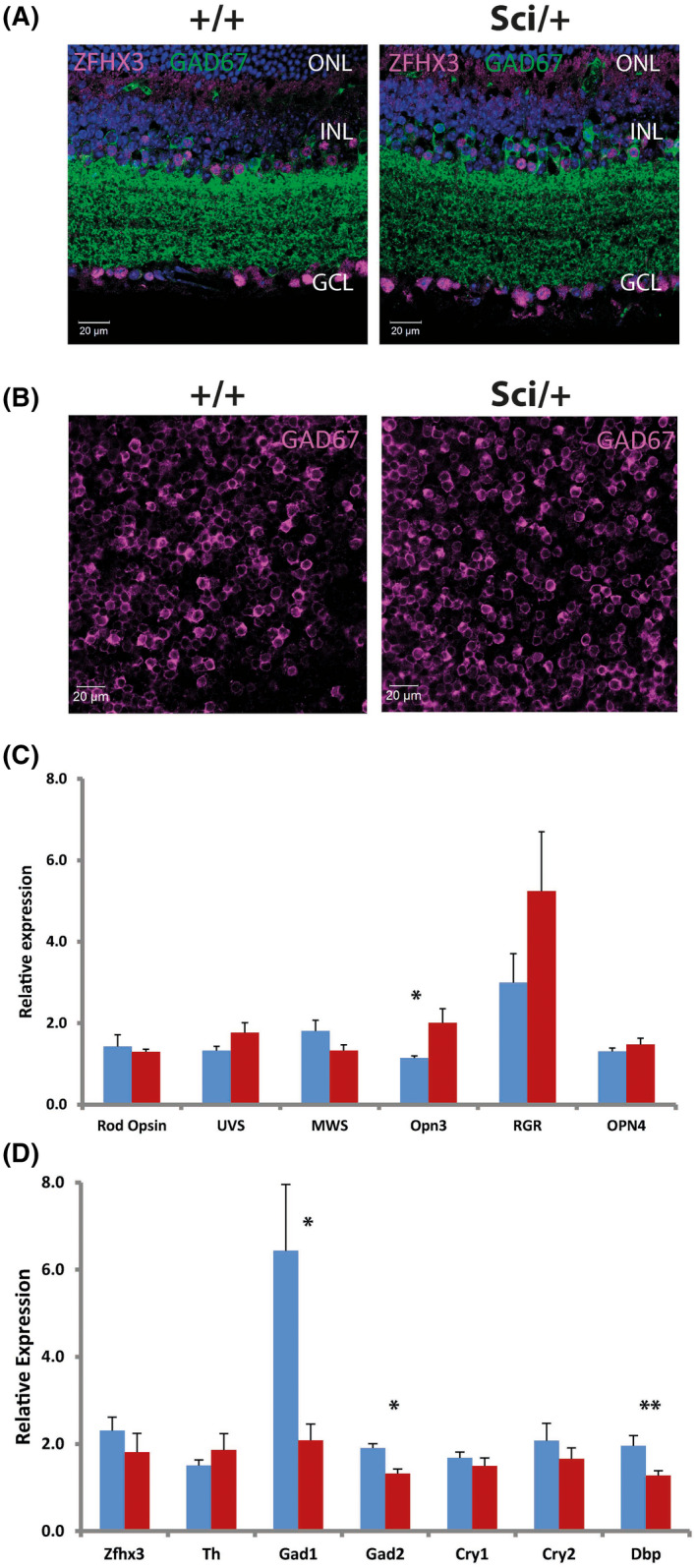
Anatomical, immunohistochemical and molecular characterization of *Zfhx3^Sci/+^* retina. A, Images showing the co‐localization of ZFHX3 and GAD67 in *Zfhx3^Sci/+^* and *Zfhx3^+/+^* retina. No overt differences in expression or localization of ZFHX3 and GAD67 were observed between *Zfhx3^Sci/+^* compared to *Zfhx3^+/+^* control retina. B, Images showing flat‐mount expression of GAD67 in *Zfhx3^Sci/+^* and *Zfhx3^+/+^* retina. DAPI nuclear counter stain is shown in blue. ONL, outer nuclear layer; OPL, Outer plexiform layer; INL, inner nuclear layer; IPL—inner plexiform layer; GCL, ganglion cell layer. C, D, Relative expression of key genes in *Zfhx3^+/+^* (blue) and *Zfhx3^Sci/+^* (red) retina (n = 6, ZT 8). Mean ± SEM (*P < .05, **P < .01)

We investigated the eyes of *Zfhx3^Sci/+^* mice for any gross anatomical abnormalities. Slit lamp and ophthalmoscope assessment did not identify any signs of abnormal eye morphologies, or other ocular conditions such as cataracts (Data not shown). Histological analysis showed that the retina of *Zfhx3^Sci/+^* mice was grossly normal, with no obvious anatomical changes or differences in retinal layer thickness detected in comparison to control mice (Data not shown). However, a significant reduction in corneal thickness was identified in *Zfhx3^Sci/+^* animals compared to controls (*P* < .0001) (Figure S1). We used fluorescence immunohistochemistry to investigate whether differences in cell identity or in expression of cell markers were evident in mutant retinas. Immunostaining with a range of antibodies recognizing key retinal cell types did not show any obvious changes in *Zfhx3^Sci/+^* retina, with normal patterns of immunostaining observed for UVS cone opsin (M and S‐cones), rhodopsin (rods), melanopsin (ipRGCs), Brn3a (major subset of RGCs), CHX10 (bipolar cells), PKCα (ON bipolar cells), calbindin (horizontal cells, and a subset of amacrine cells), Tyrosine hydroxylase (TH) (dopaminergic amacrine cells), GABA (GABAergic amacrine cells), and glycine transporter‐1 (GlyT‐1) (glycinergic amacrine cells) (Figure S2). Notably, no detectable differences in levels or localization of ZFHX3 or GAD67 protein were observed between retinas of *Zfhx3^Sci/+^* and controls (Figures 5A,B and S3).

### 3.4 Reduced expression of GABA synthetic enzyme genes in *Zfhx3^Sci/+^* retina

Given previous work implicating ZFHX3 in transcriptional regulation, we next investigated whether altered retinal expression of particular genes and proteins might account for the anomalies in light‐responsiveness found in *Zfhx3^Sci/+^* mice. Quantitative real‐time PCR (qRT‐PCR) was performed upon retinal tissue to investigate whether a subset of genes was mis‐expressed. Transcripts analyzed included a range of circadian clock genes, markers of specific retina cell types and components of key neurotransmitter signaling pathways. qPCR analysis showed no significant difference in levels of mRNA encoding rod opsin, ultra‐violet sensitive (UVS) cone opsin, middle wave‐length sensitive (MWS) cone opsin, encephalopsin (*Opn3*), retinal G protein coupled receptor (*Rgr*), melanopsin (*Opn4*), *Zfhx3*, tyrosine hydroxylase, *Pacap*, *Cry1* and *Cry2* between *Zfhx3^+/+^* and *Zfhx3^Sci/+^* animals. A significant up‐regulation of *Opn3* (*P* = .03) and significant down‐regulation of *Dbp* (*P* < .005) was observed in the *Zfhx3^Sci/+^* retina. Furthermore, although there were no evident differences in GAD67 immunolocalization in mutant retinas, a significant downregulation of both *Gad1* and *Gad2* was observed in mutant retinas (*P* < .05, Figure 5C,D).

### 3.5 Enhanced visual evoked potentials in *Zfhx3^Sci/+^* mice

**FIGURE 6:**
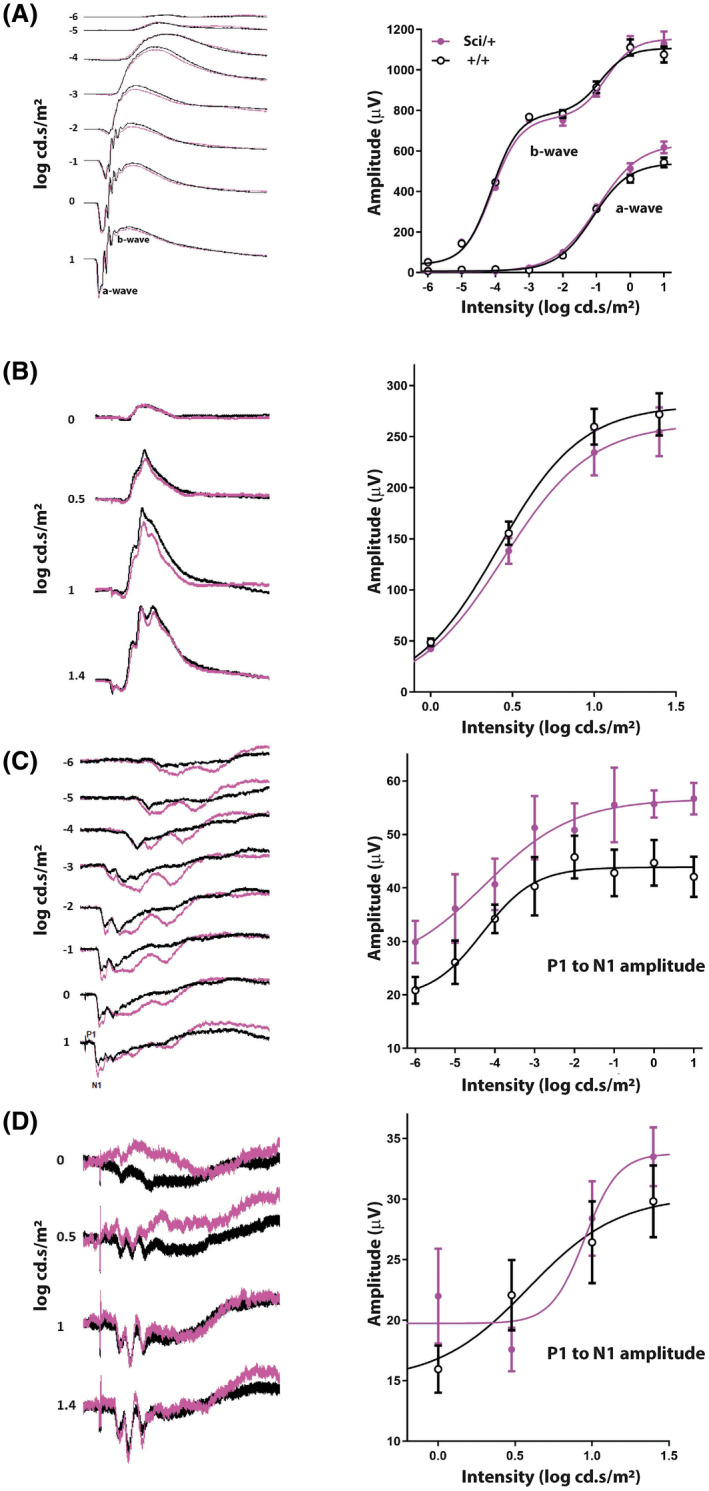
Assessment of visual pathways in *Zfhx3^+/+^* and *Zfhx3^Sci/+^* animals. A, Dark adapted ERG recordings reflecting the electrical activity of the retina. Raw data (left) and the amplitude of a‐waves and b‐waves (right) are shown across multiple light intensities. B, Light adapted ERG recordings showing raw data (left) and the amplitudes of b‐waves detected at various light intensities. C, Dark adapted VEP recordings (left) and intensity response curve effect on P1—N1 amplitude (right). D, Light adapted VEP recordings (left) and intensity response curve effect on P1 and N1 amplitude (right)

We sought to determine the electrophysiological effect of the *Zfhx3^Sci^* mutation on the retinal response to light, using electroretinography (ERG) and flash visual evoked potential (VEP) recordings (Figure 6). ERG recordings were performed under dark adapted and light adapted conditions to examine predominantly rod photoreceptor and cone photoreceptor pathway function respectively. Overt phenotypes were not detected in ERG recordings of *Zfhx3^Sci/+^* animals, with the amplitudes and sensitivities of a‐waves (indicative of photoreceptor activation) and b‐waves (downstream bipolar cell activation) not significantly different from those observed from control mice under either dark‐adapted (Figure 6A) or light‐adapted conditions (Figure 6B) across a wide range of stimulus intensities.

In addition to ERG recording, visual evoked potentials (VEPs) were recorded using electrodes placed sub‐dermally over the striate cortex in response to flash stimuli across a range of intensities (Figure 6C,D). Under dark adapted conditions a slightly larger P1 to N1 amplitude was detected in *Zfhx3^Sci/+^* mice across all light intensities (Figure 6C). These differences reached borderline significance (*P* = .0602) while a curve comparison *F*‐test indicated a significant genotype effect in the amplitude of the maximum achievable response (*P* = .0006). These differences were consistently observed in dark‐adapted animals and show a strong trend indicating that *Zfhx3^Sci/+^* mice can exhibit an exaggerated cortical response to a bright flash stimulus when compared to controls animals.

### 3.6 *Zfhx3^Sci/+^* retina shows increased sensitivity of light responses in multiple electrode array (MEA) recordings

**FIGURE 7:**
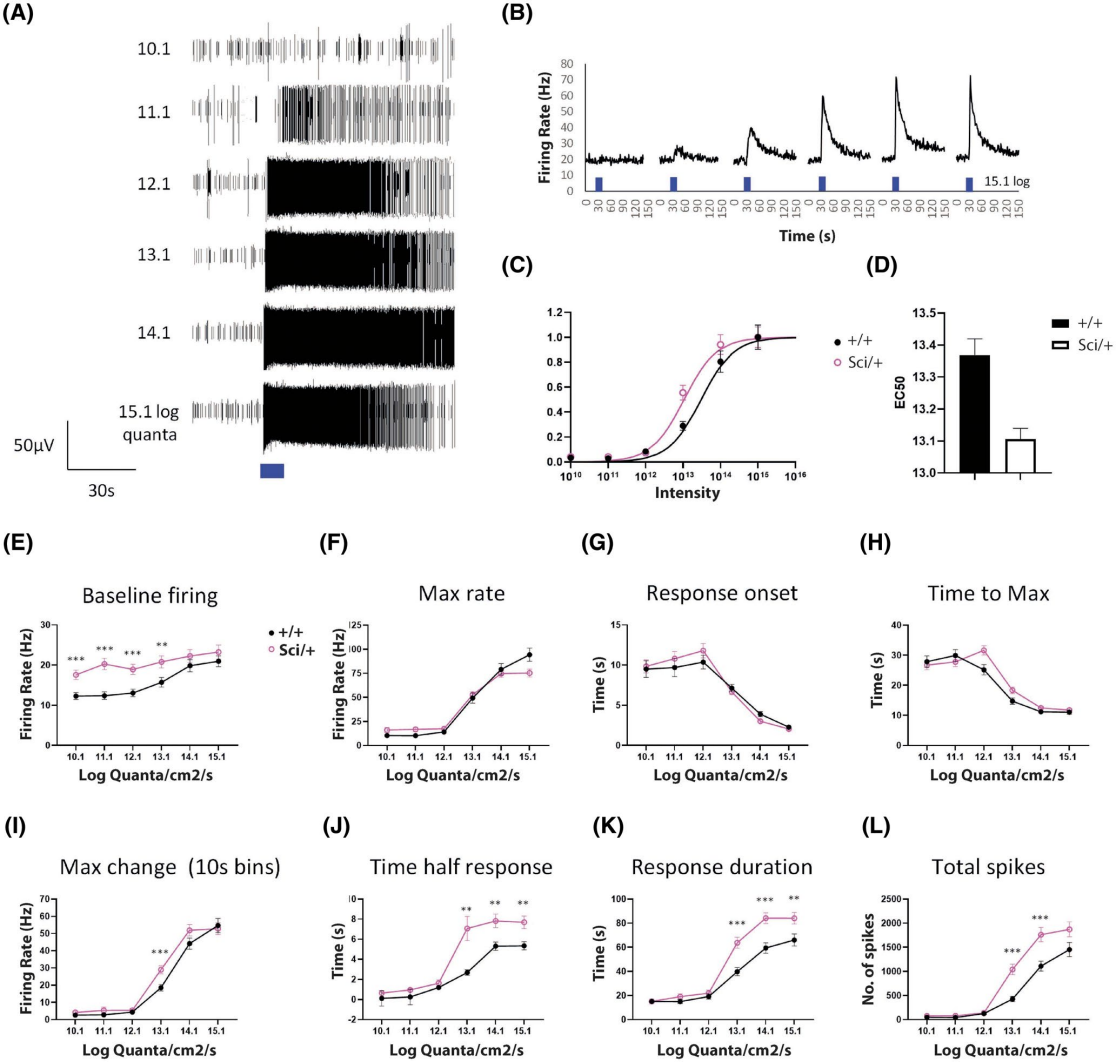
A, Raw spike data showing examples of melanopsin type responses recorded from ipRGCs stimulated with increasing intensities of 480 nm light (480 ± 10 nm, 10 seconds, ranging from 10.1‐15.1 log quanta). B, Graph showing changes in spike firing rate over time observed from an ipRGC stimulated with increasing intensities of 480 nm light. C, Intensity response curves plotted from mean normalized response amplitudes observed for each responsive electrode in *Zfhx3^+/+^* and *Zfhx3^Sci/+^* retina. D, Graph showing calculated EC50 values for melanopsin responses observed in *Zfhx3^+/+^* and *Zfhx3^Sci/+^* retina. E‐L, Graphs showing the properties of melanopsin light responses recorded from *Zfhx3^+/+^* and *Zfhx3^Sci/+^* retina. All data shown is generated from pooled analysis of individual responsive electrodes recorded from *Zfhx3^+/+^* and *Zfhx3^Sci/+^* retina (n = 96, and n = 131 electrodes, from N = 4‐5 retina). Asterisks denote the significance of post‐hoc *t*‐tests following analysis by 2‐way ANOVA (**P < .01, ***P < .001)

Given the evidence for ZFHX3 expression within ipRGCs and altered light entrainment and enhanced pupillary light responses in *Zfhx3^Sci/+^* mice, behaviors both driven by ipRGCs, we next used multiple electrode array (MEA) recordings of ex vivo retinal explants to assess light responses in *Zfhx3^Sci/+^* mice. MEA recordings were performed in normal AMES media allowing characterization of rod, cone and melanopsin driven light responses, and then in the presence of a cocktail of synaptic blockers^18^ to isolate endogenous melanopsin driven light responses of ipRGCs (Figure 7).

Construction of intensity response curves (based on data pooled from all responsive electrodes from n = 4 retinas) showed an increase in sensitivity (0.3‐0.4 log units) of melanopsin driven ipRGC light responses in *Zfhx3^Sci/+^* mice compared to control animals (2‐tailed *t*‐test, *P* = .003, Figure 7A‐D). Analysis of spontaneous ipRGC spike firing rates showed a modest but consistent increase in baseline spike firing rates (measured for the 10 seconds immediately prior to stimulation at each intensity of light) observed in *Zfhx3^Sci/+^* retina compared to controls (2‐way ANOVA, *P* < .0001, Figure 7E), however no differences in maximum firing rates were observed between *Zfhx3^Sci/+^* and wildtype retina (2 way ANOVA, *P* = .3, Figure 7F). There was also no observable difference in the rate of response onset (2‐way ANOVA, *P* = .5, Figure 7G) or time to reach maximum response (2‐way ANOVA, *P* = .052, Figure 7H) between *Zfhx3^Sci/+^* and control retina. However, the maximum amplitude of ipRGC light responses (change in spike firing rate) was moderately increased in *Zfhx3^Sci/+^* retina (2‐way ANOVA, *P* = .01), showing a difference at only moderate levels of light (12.1 log quanta) with saturation reached in both genotypes at higher intensities (Figure 7I). Significant differences were also observed for two different markers of response inactivation, including time to half response (50% response decay) (2‐way ANOVA, *P* < .0001, Figure 7J) and total duration of response (time to return to baseline firing levels) (2‐way ANOVA, *P* < .0001, Figure 7K). Combined, the increased response amplitude and increased duration of responses observed for *Zfhx3^Sci/+^* retina produces a marked elevation in the total number of spikes fired (2‐way ANOVA, *P* < .0001, Figure 7L). Collectively these results suggest an increase in sensitivity and duration of melanopsin driven light responses, resulting in an increase in total spike firing output of ipRGCs of the *Zfhx3^Sci/+^* retina compared to control retina. Notably the increased sensitivity of ipRGC responses occurred over a similar intensity range to the enhancement of pupillary light responses observed from *Zfhx3^Sci/+^* mice.

## 4 DISCUSSION

Here, we have provided a detailed report on retinal functions of the transcription factor zinc finger homeobox 3, ZFHX3, through investigation of its role in behaviors associated with visual responsiveness, its expression patterns in retina and its contribution to retinal neurophysiology. Using immunohistochemistry, we established that ZFHX3 is expressed in populations of amacrine and retinal ganglion cells in adult retina (Figures 1 and 2). By investigating retinal functions in the mutation *Zfhx3^Sci^*, we established that light responsiveness was either reduced (Figure 3), increased (Figure 4) or relatively unaffected (Figures 5 and 6). Moreover, electrophysiological recordings established that retinal ganglion cell responses showed increased sensitivity (Figure 7). Typically, as has been found for other transcription factors, the pleiotropy associated with mutations in this gene is a complex integration of its functional outputs of the diverse cell types wherein it is expressed.^5^ In this case, contributions of deficits in afferent systems (eg, retina) and of particular target tissues (eg, SCN) underlie the complex assortment of phenotypes detected. Our previous work has focused on ZFHX3's role in the SCN where dysfunctions associated with the *Zfhx3^Sci^* allele include a short free‐running period in constant darkness, a phenotype that is at least partially mediated through misregulation of several neurotransmitters, neuromodulators and their associated regulatory systems.^7^

Further investigations into the function of ZFHX3 in retina were partially based on published reports on the expression profile of the gene in developing and adult retina. Initial studies using in situ hybridization^21^ were confirmed and elaborated upon in a microarray study of developing and adult mouse retina where *Zfhx3* expression was detected at early developmental stages in amacrine cells while these expression levels were restricted in adults.^20^ Intriguingly, this pattern mirrors that seen in the CNS in general where the gene is transiently expressed at high levels in differentiating neurons but then is restricted to specific regions and clusters of cells at later developmental stages and in adults.^21^, ^22^, ^23^ Using immunocytochemistry, we expanded this study confirming that ZFHX3 expression is time‐restricted in developing amacrine cells whilst also detecting expression in RGCs (including ipRGCs) in the retinal GCL. Furthermore, we established that ZFHX3 is restricted to particular subtypes of all of these cell classes.

We propose that abnormal behavioral responses to light in *Zfhx3^Sci/+^* mice are a consequence of ZFHX3 dysfunctions not only in retinal cells but also in target regions receiving afferent input from the retina. This could explain the apparent conflicting effects seen in mutants. In dim light, the reduced ability to entrain, apparent free running and reduced or absent period lengthening may be related to a reduction in SCN responsiveness in addition to any cellular dysfunction in the inner retina. Parsons et al proposed that ZFHX3 plays a role in intercellular coupling in the SCN, and this is consistent with the splitting of activity and reduced or absent period lengthening in constant light and in the mutant's inability to re‐entrain to a light:dark cycle following constant light conditions.^7^ The reduced ability of *Zfhx3^Sci/+^* mutants to re‐entrain is an apparent contradiction to the accelerated re‐entrainment that we found when *Zfhx3* is knocked out in the adult.^8^ However, this rather points to the contrasting effects of the two mutant alleles—while the induced knockout phenotype is restricted to adult tissue, *Zfhx3^Sci/+^* is expressed from the early in developing embryo. Moreover, while the induced knockout is a null mutation, *Zfhx3^Sci/+^* is a missense mutation that expresses both gain‐ and loss‐of‐function phenotypes.^7^

Conversely, mutants displayed parameters that are indicative of increased retinal function and sensitivity. *Zfhx3^Sci/+^* mutants displayed an exaggerated pupillary constriction in response to dim light stimuli compared to litter mate control animals. This is likely to be mediated through an increased sensitivity of M1 type ipRGCs, known to control the PLR through a dedicated afferent pathway to the retinorecipient olivary pretectal nucleus.^24^ An increase in RGC sensitivity is supported by ERG and VEP data. Initial ERG responses showed normal outer retinal function in mutants, indicating normal levels of rod and cone function (normal a‐wave), and normal transmission to bipolar cells (normal b‐wave) in mutants. Given the normal sensitivity and amplitude of rod and cone driven light responses, these results suggest that any increase in retinal sensitivity is likely due to changes in retinal processing that act downstream of rod and cone photoreceptors, and acts to enhance the sensitivity of RGC responses and signals transmitted to retinorecipient areas of the brain—likely including visual and non‐image forming regions. In support of this interpretation, altered VEP responses in *Zfhx3^Sci/+^* animals indicate that there is an increased or “over‐exaggerated” cortical response to photic stimuli compared to *Zfhx3^+/+^* littermates. Similarly, MEA recordings of retinal explants showed enhanced light responses recorded from ipRGCs in *Zfhx3^Sci/+^* mice where, notably, the increased sensitivity of ipRGC responses occurred over a similar intensity range to the enhancement of pupillary light responses.

Adult patterns of ZFHX3 expression in retina would suggest that the effects of the *Zfhx3^Sci^* allele are mediated through dysfunctions either in GABAergic amacrine cells and/or in subclasses of RGCs including ipRGCs. Furthermore, the mutant allele has previously been shown to mediate its effects through the misregulation of disparate components of multiple neurotransmitter systems in SCN.^7^ An initial investigation of a number of neurotransmitter candidate systems in retinal tissue uncovered significant reductions in retinal *Gad1* and *Gad2* mRNA expression. These genes encode the GABA synthetic enzyme glutamic acid decarboxylase (GAD), and reduced expression should result in reduced GABA production and a consequential decrease in GABA signaling. Although no great differences in the number of GABAergic amacrine cells or in GAD immunoreactivity were detected in *Zfhx3^Sci/+^* mutants, it is still reasonable to consider that disturbed GABAergic amacrine cell activity may underlie a disinhibition of some retinal ganglion cell functions in *Zfhx3^Sci/+^* mice. This is manifest in the increased baseline activity and enhanced sensitivity of retinal light responses to weaker stimuli in *Zfhx3^Sci/+^* mutants. A dis‐inhibition of the inner retina may in turn mediate the observed effects on the pupillary response. Indeed, GABA is also documented to be involved in manipulating the circadian responses of the inner retina while GABA(A) receptors have been shown to modulate the responsiveness of the circadian system to light.^25^

## 5 CONCLUSION

This is the first study to implicate ZFHX3 in photic processing and in modulation of the pupillary response. The findings presented in this study demonstrate that *Zfhx3^Sci/+^* is a useful mouse model displaying both circadian and ophthalmological phenotypes. *Zfhx3^Sci/+^* mice present with a rare pupillary reflex phenotype; over‐constriction in response to scotopic irradiances, and is therefore a useful model of enhanced photic sensitivity. Molecular characterization has shown that ZFHX3 is expressed in specific layers of the adult retina, and the mutation has an effect on retinal neurotransmission, likely at the level of GABAergic amacrine cell signaling.

ZFHX3 is therefore likely a critical component in modulating the pupillary response, in particular under scotopic irradiances, and participates in non‐imaging forming visual pathways in addition to its previously characterized role in circadian rhythm generation. Collectively, these data suggest a role for ZFHX3 in the retina, seemingly acting downstream of rods and cones to modulate the sensitivity of ipRGC light responses and photic entrainment pathways. Phenotypes observed in mutant mice are likely mediated either by a reduction of inhibitory signaling from GABAergic amacrine cells or by direct modulation of ipRGC function. Meanwhile, reduced light responsiveness is more likely related to the effects of mutant gene expression in retinal brain targets. These data provide a new mechanism by which ZFHX3 likely influences entrainment pathways and highlights the need to consider retinal phenotypes in animal models with altered circadian function that have been attributed to central SCN dysregulation. Further work remains to fully elucidate the mechanism by which ZFHX3 modulates the pupillary response in mammals through GABA signaling and the functional contribution of ZFHX3 in the melanopsin signaling cascade.

## CONFLICT OF INTEREST

The authors declare no competing interests.

## AUTHOR CONTRIBUTIONS

S. Hughes, M.W. Hankins, S.N. Peirson, G. Banks and P.M. Nolan designed the research. S. Hughes, J.K. Edwards, A.G. Wilcox, C.A. Pothecary, A.R. Barnard and R. Joynson performed research. G. Joynson contributed reagents or tools. S. Hughes, J.K. Edwards and G. Banks analyzed data. S. Hughes, A.R. Barnard, S.N. Peirson, G. Banks and P.M. Nolan wrote the paper.

## Supporting information

Fig S1

Fig S2

Fig S3

Text S1
