# Supplementary figures and images for: Zfhx3 modulates retinal sensitivity and circadian responses to light
Source: FASEB J. 2021 Aug 12;35(9):e21802. doi: 10.1096/fj.202100563R (PMC9292409; doi:10.1096/fj.202100563R)

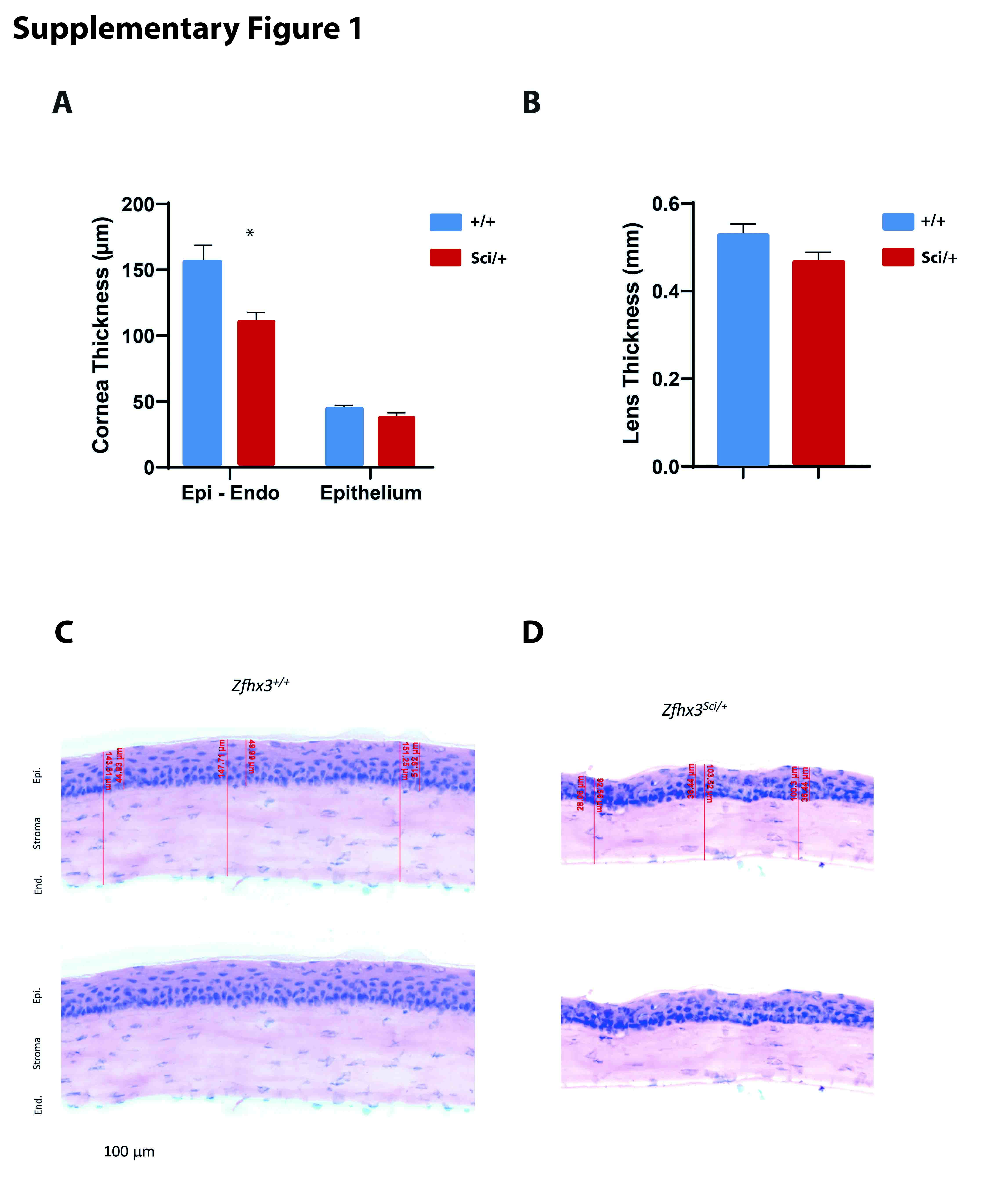

Supplement: Supplementary file 1 — Fig S1 [file FSB2-35-0-s003.jpg]

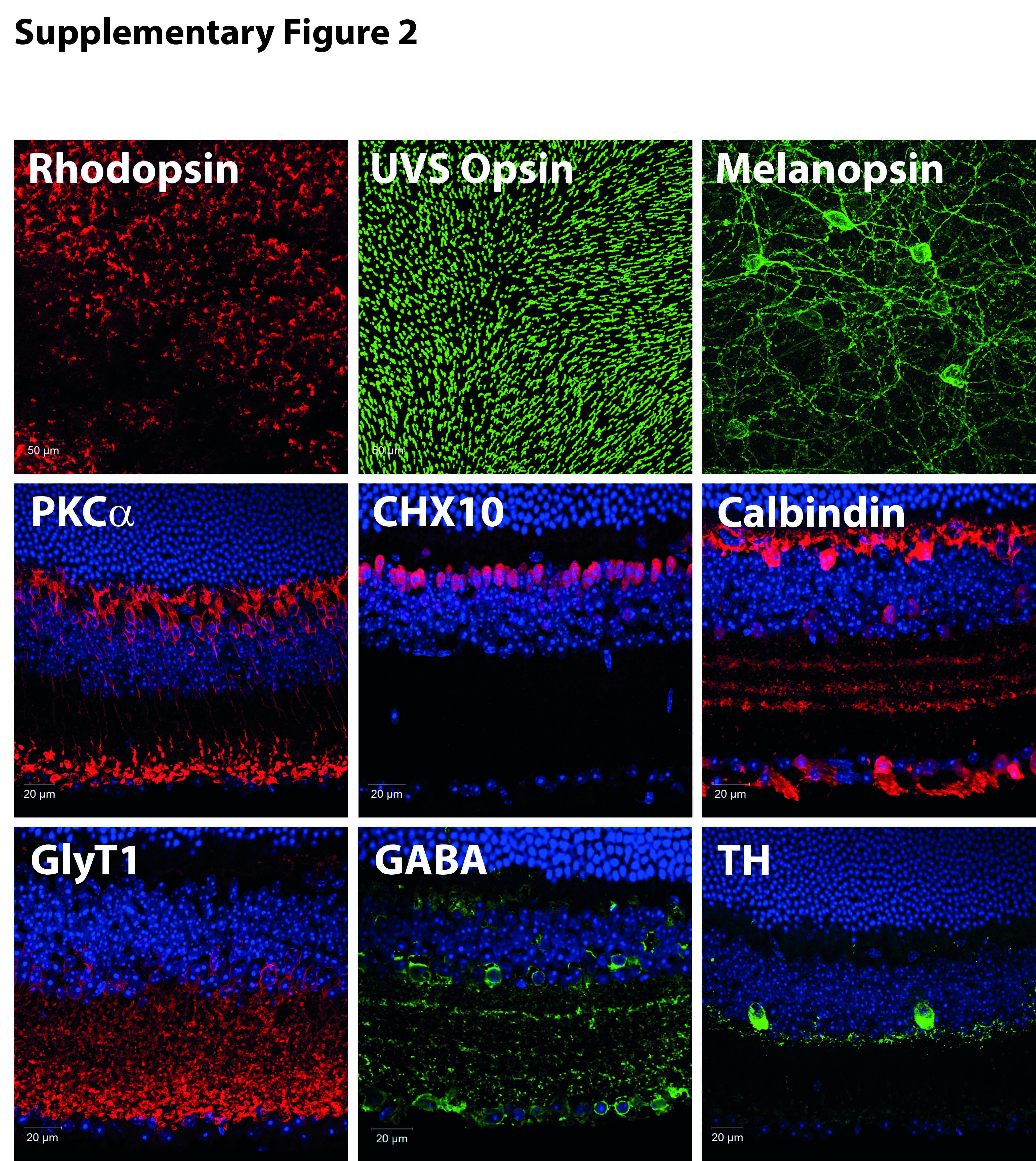

Supplement: Supplementary file 2 — Fig S2 [file FSB2-35-0-s002.jpg]

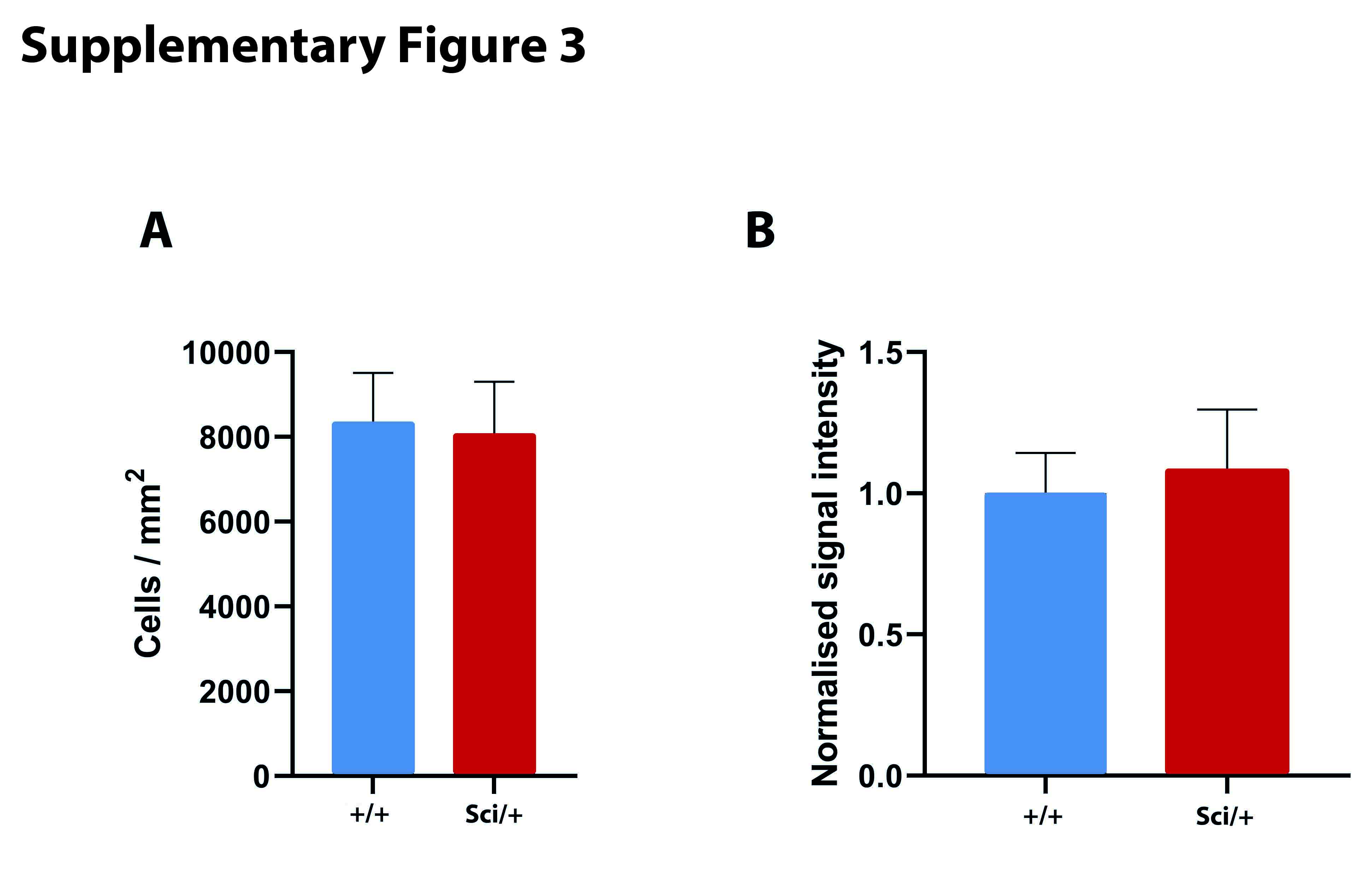

Supplement: Supplementary file 3 — Fig S3 [file FSB2-35-0-s004.jpg]
