# Supplementary material for: Zfhx3 modulates retinal sensitivity and circadian responses to light
Source: FASEB J. 2021 Aug 12;35(9):e21802. doi: 10.1096/fj.202100563R (PMC9292409; doi:10.1096/fj.202100563R)
Supplement: Supplementary file 4 — Text S1 [file FSB2-35-0-s001.docx]

**Supplemental Information**

***Zfhx3* modulates retinal sensitivity and circadian responses to light.**

**Short title:** *Zfhx3* modulates retinal function.

Steven Hughes^1^, Jessica K Edwards^2^, Ashleigh Wilcox^2^, Carina A Pothecary^1^, Alun R. Barnard^3^, Russell Joynson^2^, Greg Joynson^2^, Mark W Hankins^1^, Stuart N Peirson^1^, Gareth Banks^2^ and Patrick M Nolan^2^

**Supplementary Table 1. Primary and secondary antibodies**

| **Target** | **Host** | **Source** | **Dil’n** | **Secondary antibody (1:200)** |
| --- | --- | --- | --- | --- |
| ZFHX3 | Rabbit | Custom generated | 1:400 | Donkey anti-rabbit Alex 568 |
| GAD67 | Goat | MAB5406, Merck | 1:250 | Donkey anti-goat Alex 488 |
| Brn3a | Goat | sc-31985, Santa Cruz Biotech | 1:1000 | Donkey anti-goat Alex 488 |
| βgal | Chicken | Ab9361, Abcam | 1:500 | Donkey anti-chicken Alex 488 |
| eYFP | Chicken | GFP-1020, AVES Labs | 1:1000 | Donkey anti-chicken Alex 488 |
| UVS | Goat | sc-14363, Santa Cruz Biotech | 1:1000 | Donkey anti-goat Alex 488 |
| Rhodopsin | Mouse | ID4, Gift Jill Cowing | 1:3000 | Donkey anti-mouse Alex 568 |
| Melanopsin | Rabbit | UF006, Advanced Targeting Systems | 1:2500 | Donkey anti-rabbit Alex 488 |
| Calbindin | Rabbit | ab11426, Abcam | 1:1000 | Donkey anti-rabbit Alex 568 |
| CHX10 | Sheep | ab16141, Abcam | 1:400 | Donkey anti-sheep Alex 568 |
| PKCα | Rabbit | ab32376, Abcam | 1:1000 | Donkey anti-rabbit Alex 568 |
| TH | Chicken | ab76442, Abcam | 1:1000 | Donkey anti-chicken Alex 488 |
| GlyT-1 | Goat | AB1770, Millipore | 1:1000 | Donkey anti-goat Alex 568 |
| GABA | Mouse | GB-69, Sigma | 1:250 | Donkey anti-mouse Alex 488 |

**Supplementary Table 2. RT-PCR Primer Sequences**

| **Primer Name** | **Sequence 5’ to 3’** |
| --- | --- |
| **Rpli3a FW** | GGAAGCGGATGAATACCAAC |
| **Rpli3a Rev** | GGATCCCATCCAACACCTT |
| **Dbp FW** | GAGCCTTCTGCAGGGAAACA |
| **Dbp Rev** | GCCTTGCGCTCCTTTTCC |
| **Cry1 FW** | GCTATGCTCCTGGAGAGAACGT |
| **Cry1 Rev** | TGTCCCCGTGAGCATAGTGTAA |
| **Cry2 FW** | TGACCTAGACAGAATCATCGAACTG |
| **Cry2 Rev** | GGCTGATGAGGGCCTGAA |
| **Per1 FW** | CCCCTGCCTCCCAGTGA |
| **Per1 Rev** | CTGAAAGTGCATCCTGATTGGA |
| **Per2 FW** | AGCTACACCACCCCTTACAAGCT |
| **Per2 Rev** | GACACGGCAGAAAAAAGATTTCTC |
| **Clock FW** | TGTCTCAAGCTGCAAATTTACCA |
| **Clock Rev** | TTTAGATGCTGCATGGCTCCTA |
| **Bmal1 FW** | CCGTGCTAAGGATGGCTGTT |
| **Bmal1 Rev** | TTGGCTTGTAGTTTGCTTCTG |
| **Reverba FW** | CGTTCGCATCAATCGCAACC |
| **Reverba Rev** | GATGTGGAGTAGGTGAGGTC |
| **Zfhx3 FW** | CCAATAGCCTGGAGAAGCTG |
| **Zfhx3 Rev** | AGTTGCACAGGACACAGTGG |
| **Rod opsin FW** | TGTTCCTGCTCATCGTGCTGG |
| **Rod Opsin Rev** | GGAAGTTGCTCATCGGCTTGC |
| **UVS FW** | TCTTCACAGTCTTCATCGCCAGC |
| **UVS Rev** | GTTCAAAAGCCAGGAAAGCCAATG |
| **MWS FW** | ATGGTGGTGGTGATGGTCTTCG |
| **MWS Rev** | TGTCTTGGAGGTGCTGGAAAGTTC |
| **Encephalopsin FW** | AAGGTGACTCCGAACAGGGATACC |
| **Encephalopsin Rev** | GCTGGTGCTGCTTCTCTACTCCAAG |
| **RGR FW** | CGAGGGGTGACAGAAACTTCATCAG |
| **RGR Rev** | CCAGCCAAGCAGCAGCATTC |
| **Tyrosine Hydroxylase FW** | GATTGCAGAGATTGCCTTCC |
| **Tyrosine Hydroxylase Rev** | GGTAGCAATTTCCTCCTTTGTG |
| **PACAP FW** | CCCTGCTGGTGTATGGGATA |
| **PACAP REV** | TGGTCGTAAGCCTCGTCTTC |
| **GAD2 FW** | AGGTGGCCCAAAAGTTCAC |
| **GAD2 REV** | CCGGAGTCTCCATAGAGCAG |
| **GAD1 FW** | ACTGGGCCTGAAGATCTGTG |
| **GAD1 Rev** | CAGGAAAGCAGGTTCTTGGA |

**Supplementary Figure 1. Corneal thickness in** ***Zfhx3^Sci/+^* and *Zfhx3^+/+^* mice.** (A) Comparison of cornea and lens thickness in *Zfhx3^+/+^* (black, n=3) and *Zfhx3^Sci/+^* (white, n=3) mice. (B) Images of H&E stained cornea showing significant differences in corneal thickness between *Zfhx3^+/+^* wildtype *and Zfhx3^Sci/+^* mutant mice. Mean + SEM (*p<0.0001).

**Supplementary Figure 2. Retinal marker expression is unaffected in *Zfhx3^Sci/+^* retina.** Images showing the expression of key retinal markers in the retina of *Zfhx3^Sci/+^* animals. Overall the retina of *Zfhx3^Sci/+^* mice appeared grossly normal, with no obvious differences in the number or distribution of key retina cell types, or levels of protein expression detected for UVS cone opsin (M and S-cones), rhodopsin (rods), melanopsin (ipRGCs), Brn3a (RGCs), CHX10 (bipolar cells) and PKCα (ON bipolar cells), calbindin (horizontal cells, and a subset of amacrine cells), Tyrosine hydroxylase (TH) (dopaminergic amacrine cells), GAD67 (GABAergic amacrine cells), GABA, and glycine transporter-1 (GLYT-1) (glycinergic amacrine cells).

**Supplementary Figure 3. GAD67 immunoreactivity is unchanged in *Zfhx3^Sci/+^* retina.** (A) Graphs showing no difference in the density of GAD67 positive amacrine cells in *Zfhx3^Sci/+^* and *Zfhx3^+/+^* retina. (B) Analysis of mean pixel intensity using standard settings in ImageJ shows no overall difference in levels of GAD67 immunoreactivity between *Zfhx3^Sci/+^* and *Zfhx3^+/+^* mice. Analysis based on n=4 images collected from N=3 retina for each genotype.
